# Preparation, modification, and clinical application of porous tantalum scaffolds

**DOI:** 10.3389/fbioe.2023.1127939

**Published:** 2023-04-04

**Authors:** Xinyi Wang, Ke Zhou, Yada Li, Hui Xie, Benjie Wang

**Affiliations:** Affiliated Zhongshan Hospital of Dalian University, Dalian, China

**Keywords:** porous Ta, preparation, surface modification, clinical application, porous tantalum scaffolds

## Abstract

Porous tantalum (Ta) implants have been developed and clinically applied as high-quality implant biomaterials in the orthopedics field because of their excellent corrosion resistance, biocompatibility, osteointegration, and bone conductivity. Porous Ta allows fine bone ingrowth and new bone formation through the inner space because of its high porosity and interconnected pore structure. It contributes to rapid bone integration and long-term stability of osseointegrated implants. Porous Ta has excellent wetting properties and high surface energy, which facilitate the adhesion, proliferation, and mineralization of osteoblasts. Moreover, porous Ta is superior to classical metallic materials in avoiding the stress shielding effect, minimizing the loss of marginal bone, and improving primary stability because of its low elastic modulus and high friction coefficient. Accordingly, the excellent biological and mechanical properties of porous Ta are primarily responsible for its rising clinical translation trend. Over the past 2 decades, advanced fabrication strategies such as emerging manufacturing technologies, surface modification techniques, and patient-oriented designs have remarkably influenced the microstructural characteristic, bioactive performance, and clinical indications of porous Ta scaffolds. The present review offers an overview of the fabrication methods, modification techniques, and orthopedic applications of porous Ta implants.

## 1 Introduction

Tantalum (Ta) has been used in clinical practice as a surgical suture, bone fixation component, bone implant, vascular stent coating, and medical imaging contrast agent since its discovery because of its high biological affinity and superior physicochemical and biological properties (Yang et al., 2020a). Ta possesses excellent biocompatibility, corrosion resistance, mechanical ductility, osteoconductivity, osteoinductivity, and vascular inductivity. Furthermore, it has a high affinity for oxygen and tends to form a self-passivation surface oxide layer (Ta_2_O_5_), thus allowing the formation of bone-like apatite coating ^[^ (Wang et al., 2016a)^,^ (Rupérez et al., 2015)^]^. Moreover, Ta oxide coatings exhibit remarkable antibacterial properties (Chang et al., 2014). These features make Ta ideal for orthopedic applications.

In the 1990s, porous Ta scaffolds were prepared through chemical vapor deposition (CVD), and their structures and properties were analyzed (Yang et al., 2020b). Porous Ta possesses a completely interconnected structure, high osteoconductivity, and low elastic modulus compared with dense Ta. Because the elastic modulus of porous Ta is equivalent to that of human cancellous bone, stress load is evenly distributed on a porous Ta implant, thereby minimizing the risk of dissolution around the prosthesis and implant failure caused by stress shielding. Furthermore, porous Ta has a high friction coefficient for the bone, and thus, it exhibits high stability as an implant. Porous Ta scaffolds facilitate the attachment, proliferation, differentiation, and mineralization of osteoblasts, leading to better osteogenesis and osteointegration *in vivo* (Smith et al., 2014; Wang et al., 2015a).

Over the past 2 decades, porous Ta has been clinically used to repair defective bone tissues and treat various bone diseases. These clinical applications include hip/knee arthroplasty, spinal fusion, and femoral head necrosis treatment (Gao et al., 2021). Emerging manufacturing technologies, surface modification, and clinical applications have substantially eased manufacturing upgrades, structural and performance optimization, and application of Ta scaffolds. For instance, additive manufacturing (AM) has led to the production of patient-specific and anatomically matched Ta implants with well-designed porous structures. Levine et al. (Russell Levine et al., 2006) and George et al. (George, 2018) investigated the performance and clinical application, respectively, of porous Ta scaffolds produced through CVD. Liu et al. (Liu et al., 2015a) summarized the usefulness of porous Ta scaffolds in dental applications. Qian et al. (Hu et al., 2020) reviewed the physicochemical, cellular, animal, and clinical studies of porous Ta scaffolds prepared through AM. Han et al. (Han et al., 2019) compared the progress of porous Ta scaffolds and porous Ti-based scaffolds in orthopedics field.

The aforementioned studies have primarily summarized certain characteristics of porous Ta scaffolds; however, their properties have not been systematically examined. The present review summarizes the latest advances in the manufacturing, modification, and orthopedic applications of these scaffolds, providing a comprehensive reference for porous Ta-based implants.

## 2 Mechanical properties

Bone implant materials used to replace human bone tissue should fulfill certain conditions. First, they must have appropriate stiffness and compressive strength to provide support for the joints and a mechanical environment conducive to bone tissue regeneration for the implant because too high stiffness and compressive strength can reduce the load required for new bone formation. Second, the implant materials should possess the ability to resist fatigue fracture. Finally, the elastic modulus of the implant material should match the elastic modulus of the human bone to avoid stress shielding, which may cause implantation failure. Bone tissue grows normally only when it is subjected to an appropriate mechanical load. Insufficient force will cause bone absorption, whereas excessive stress can destroy the bone tissues (Ao et al., 2022).

### 2.1 Compressive strength

Bone tissue is sensitive to stress, and its growth is strongly associated with the stress of the surrounding bone tissue after implantation. In healthy mammals, the cortical lamellar bone can withstand a threshold of ultimate strength or fracture strength of approximately 120 MPa (or strain exceeding 25,000 με). The stress threshold for bone resorption ranges from 1 to 2 MPa (or strains less than 50–100 με), resulting in decreased bone stiffness and density. The stress threshold for bone growth is 20 MPa (or strains exceeding 1,000–1,500 με), which further results in increased bone strength. When the stress threshold of fatigue damage is 60 MPa (or the strain exceeds 3,000 με), the corresponding bone tissue is more prone to damage. Therefore, the compressive strength of the prepared porous tantalum implants is sufficient to meet the reconstruction of the mechanical function of bone tissue. Table 1 shows the results of porous tantalum compression experiment, indicating that the differences in compressive strength may be attributed to the differences in the structure and process of porous structures. The compressive strength of porous tantalum scaffolds is negatively correlated with porosity. Therefore, future studies should focus on the design of reasonable pore size or porosity to balance the compressive strength of porous tantalum scaffolds.

**TABLE 1:** Preparation technology, porosity, and mechanical properties of porous tantalum.

Material	Manufacturing method	Porosity/%	Elastic modulus/GPa	Compressive strength/MPa	Compressive yield strength/MPa	Reference
Cancellous bone		50–90	0.01–3.0		2–12	Yang et al. (2020a)
Unalloyed Ta(F560)			185		138–345	Zhou and Zhu (2013)
Porous Ta	CVD	75–85	2.3 ± 3.9	50–70	35–51	Zhou and Zhu (2013)
	PM	66.7	2.21 ± 0.16	61.5 ± 4.5		Rupérez et al. (2015)
		50	1.17 ± 0.08	34.12 ± 3.67		Xie et al. (2019)
		60	0.48 ± 0.03	20.01 ± 2.59		Xie et al. (2019)
		70	0.14 ± 0.03	8.57 ± 1.43		Xie et al. (2019)
	LENS	27–55	1.5–20			Hu et al. (2020)
	SLM	79.7 ± 0.2	1.22 ± 0.07	3.61 ± 0.4	12.7 ± 0.6	Wang et al. (2020)
	SLM	68.3 ± 1.1	2.34 ± 0.2	78.54 ± 9.1		Ma et al. (2016)

### 2.2 Fatigue strength

Bone implants can bear high cyclic loads *in vivo* (HEDAYATI et al., 2016); therefore, the fatigue performance of porous tantalum scaffolds should also be considered in the design process. Ghouse et al. (GHOUSE et al., 2018) compared the high periodic fatigue strength of four metal alloys (CP-Ti, Ti-6Al-4VELI, Ta, and Ti-30Ta), and the results showed that porous tantalum and titanium alloys had the highest fatigue strength under the same stiffness, which was 8% higher than that of CP-Ti and 19% higher than that of Ti-6Al-4VELI. At the same time, the fatigue strength of porous materials could be increased by 7%–8% by optimizing the process parameters of AM preparation. The study by Wauthle et al. (AMIN-YAVARI et al., 2013) also found that the fatigue strength of porous tantalum was significantly higher than that of porous titanium alloy (Ti-6Al-4VELI) (7.35 MPa vs 4.18 MPa) after 106 cycles. Due to its high ductility, the former allowed more plastic deformation and reduced the generation and diffusion of cracks (RITCHIE, 1999).

### 2.3 Elastic modulus

In the human body, the porosity of cortical bone is 3%–5%, and the elastic modulus is 7–30 Gpa; the porosity and elastic modulus of cancellous bone are 50%–90% and 0.01–3.0 Gpa, respectively (ZHANG et al., 1999). The elastic modulus of solid tantalum is 185 GPa, which is far higher than that of bone tissue, while the elastic modulus of porous tantalum is 2.3–30 GPa and the porosity is 27%–85% (WEI et al., 2016), thereby providing more variable space compared with cortical bone or spongy bone. According to the data in Table 1, the elastic modulus is also affected by the processing technology. Zhou et al. (ZHOU et al., 2017) determined the effect of energy density and scanning speed in selective laser melting (SLM) process on product density and used optimized SLM technology to prepare tantalum samples with the highest density of 96.92%. The microhardness increased from 120 HV to 445 HV. The tensile strength increased from 310 MPa to 739 MPa. Compared with those of powder metallurgy products, the mechanical properties were increased by more than two times. The elastic modulus of a porous structure is not only affected by its preparation technology and related parameters but also by its pore characteristics. Through numerical simulation, it is found that in porous structures, cubic hole scaffolds have higher structural modulus than inclined hole scaffolds, and the elastic modulus of porous scaffolds is negatively correlated with porosity (WIEDING et al., 2014).

## 3 Preparation of porous Ta

Ta is a refractory metal having a melting point of up to 2,980°C. Theoretically, the method for preparing porous Ta materials is the same as that for preparing other porous refractory metals. Nevertheless, Ta can react with C and O at high temperatures, and therefore, limited processing methods are available for porous Ta materials (Van Steenkiste and Gorkiewicz, 2004). The reported methods mainly include CVD, powder metallurgy (PM), and AM.

### 3.1 Foam impregnation

The aqueous solution of polyvinyl alcohol (PVA) is used as a binder, to which the Ta powder is added for obtaining a slurry of appropriate viscosity and fluidity. A polyurethane foam with three-dimensional connected pores is used as the porous support. After compression and exhaust, the foam is placed in the slurry, and the foam body is repeatedly compressed. The elastic expansion property of polyurethane facilitates the absorption of the slurry. After impregnation, the surplus slurry in the foam is removed to obtain the green body. This green body is vacuum dried. Finally, the dried green body is sintered to obtain the porous Ta (Xu and Jiang, 2016).

The PVA concentration greatly influences slurry viscosity, which in turn affects the porosity of porous Ta. Porous Ta prepared through foam impregnation is disadvantageous because pore plugging formed inevitably during slurry impregnation affects the three-dimensional interconnected pore structure and hinders the complete bone tissue growth. Therefore, this method is rarely used.

### 3.2 Powder metallurgy

**FIGURE 1:**
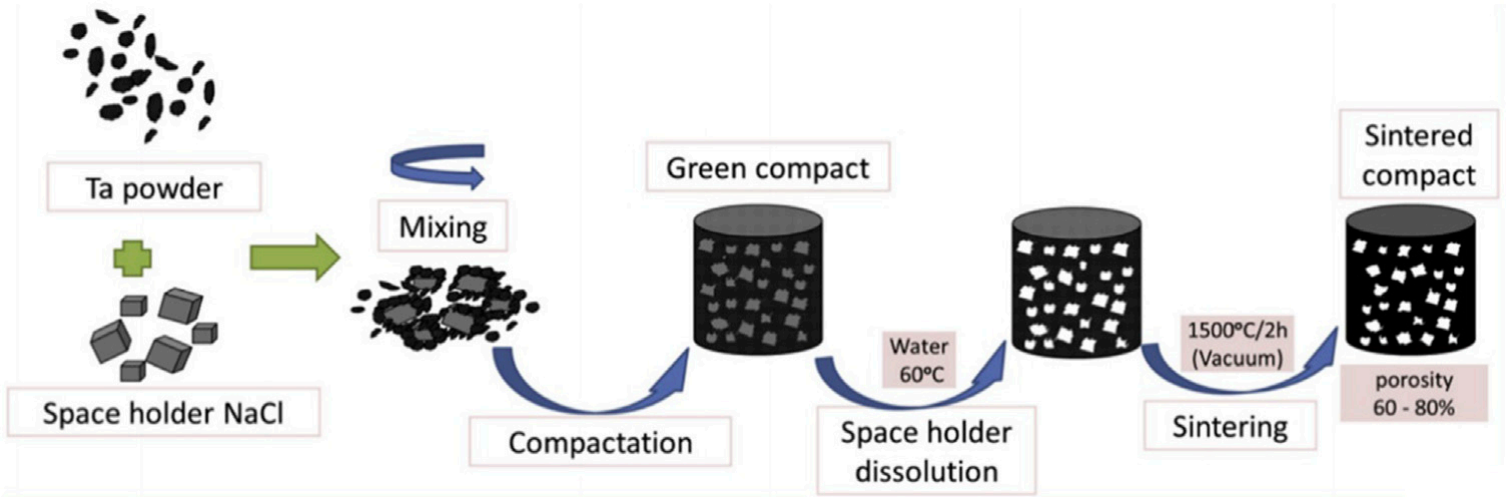
Schematic of the powder metallurgy technique (Rupérez et al., 2015). *Copyright © 2015 Published by Elsevier Ltd.*

PM is a less-cutting or no-cutting material processing method that offers unique advantages in preparing porous metals having complex structures. The main steps are illustrated in Figure 1. First, Ta powders and space-holding particles are homogenously mixed. This mixture is compacted under an appropriate pressure (350 or 450 MPa), which is applied using a uniaxial hydraulic press with a 10-mm-diameter die. The green compact obtained is then dissolved in distilled water generally maintained at 60°C to ensure a quick dissolution process, complete removal of the space-holding particles, and formation of a porous structure. Lastly, the porous sample is oven-dried for 2 h and sintered at 1,300°C–2000°C in vacuum to produce porous Ta scaffolds (Wang et al., 2015b; Rupérez et al., 2015; Raja Sukumar et al., 2019; Wang et al., 2020).

Zhou et al. (Zhou and Zhu, 2013) first mixed glutinous rice flour and NaCl particles to produce spherical particles for use as a pore-forming agent. After isostatic pressure treatment, this agent was evenly mixed with Ta powder to obtain raw billet. The billet was sintered at 1800°C, and a preliminary spongy porous Ta sample was produced after removing the glutinous rice flour. To remove NaCl, the sample was placed in circulating water, and the final porous Ta scaffolds were fabricated. The as-prepared scaffolds exhibit a connected pore structure, with a pore size of 100–400 mm, compressive strength of 50.3 ± 0.5 MPa, and elastic modulus of 2.0 ± 0.3 GPa, which are comparable to those of the cancellous bone, and thus, they can minimize the stress shielding effect.

Notably, numerous closed pores are inevitably formed during PM because of the inherent limitation of this approach. Thus, the pores of Ta scaffolds fabricated through PM have lower interconnectivity than those of scaffolds fabricated through CVD. This hinders the enhancement of osteoconductivity *in vivo* of Ta scaffolds fabricated through PM. Despite extensive material, mechanical, *in vitro*, and preclinical animal studies on PM-generated porous Ta scaffolds, their clinical applications are limited.

### 3.3 Chemical vapor deposition

**FIGURE 2:**
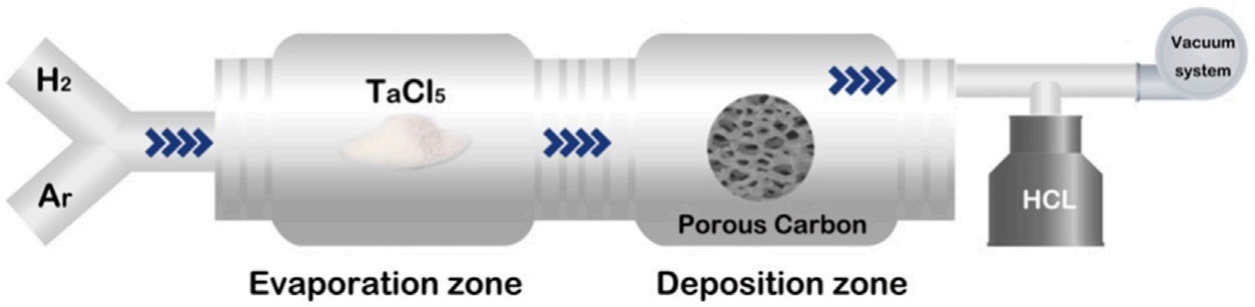
Schematic of the CVD system.

CVD is a mainstream commercial manufacturing method for porous Ta and was invented by Implex Company, which has now been taken over by Zimmer. Porous Ta implants are made through the pyrolysis of porous thermosetting polymers, which confer 98% porosity and form a low-density carbon skeleton with a repeating dodecahedral array. Pure Ta is deposited on the support through chemical vapor infiltration. For example, porous carbon is produced by pyrolysis of the polyurethane foam precursor, which leads to a glassy pyrolytic carbon skeleton with a spongy porous structure. Subsequently, commercially pure Ta serves as the raw material, and CVD is adopted to allow the pure Ta to react with Cl_2_ and generate gaseous TaCl_5_. Next, H_2_ is used to reduce Ta from TaCl_5_ and deposit Ta onto the carbon skeleton to form a unique porous structure (Xie et al., 2019). A typical CVD system is schematized in Figure 2.

The porous Ta produced using the aforementioned method maintains a skeleton with a spongy porous structure, comprising crisscross grids and pores. The grids are arranged in multiple dodecahedral structures. Thickness of Ta coating on the carbon skeleton is between a few micrometers to several hundred micrometers; this thickness is achieved by adjusting the reaction time. The Ta coating thickness is generally 40–60 μm, with the pore size being approximately 400–600 μm and the porosity being 75%–85%. These values indicate that the coating meets the clinical requirements in terms of mechanical properties and for bone penetration.

Ma et al. (Ma et al., 2016; Ma et al., 2020a) generated porous Ta scaffolds with a bone trabecular structure on the surface of a porous SiC scaffold through CVD. In such cases, the Ta coating thickness is linearly related to the deposition time and is accurately regulated from a few micrometers to hundreds of micrometers. Consequently, the scaffold porosity and pore size are customized, thereby resulting in various mechanical performances and in turn meeting the demand of different implantation sites. The elastic modulus of porous Ta scaffolds obtained through the aforementioned process is 1–30 GPa, and the corresponding compressive strength is 10–200 MPa (Ma et al., 2020a).

Despite the widespread use of CVD-manufactured Ta scaffolds in bone defect repair and bone disease treatment, more advanced manufacturing techniques are required because of the following reasons. First, CVD is a conventional, high-cost manufacturing process. Second, fabrication of patient-specific and anatomically matched shapes is difficult. Furthermore, CVD lacks designability and controllability of porous structures.

### 3.4 Additive manufacturing

Customized patient-specific bone implants are required for proper matching of anatomical shapes at different anatomical sites, and personalized treatment is the current trend in orthopedic clinical practice. AM, a powerful and multi-functional processing technique, also known as rapid prototyping technology or 3D printing (Hu et al., 2020), has emerged in the past few years. It is based on 3D model data and adopts layer-by-layer superposition. AM techniques include powder bed laser fusion, powder bed electron fusion, selective laser sintering, directed energy deposition, direct metal deposition, direct metal printing, fused deposition modeling, direct metal writing, and binder jetting (Yang et al., 2020c). Highly customized porous implants with complex geometric shapes that match real anatomical shapes can be manufactured through AM. Moreover, the porosity of such implants can be designed and adjusted to regulate the compressive strength and elastic modulus and avoid stress shielding (Wauthle et al., 2015a; Zhao et al., 2018a; Amit et al., 2019).

**FIGURE 3:**
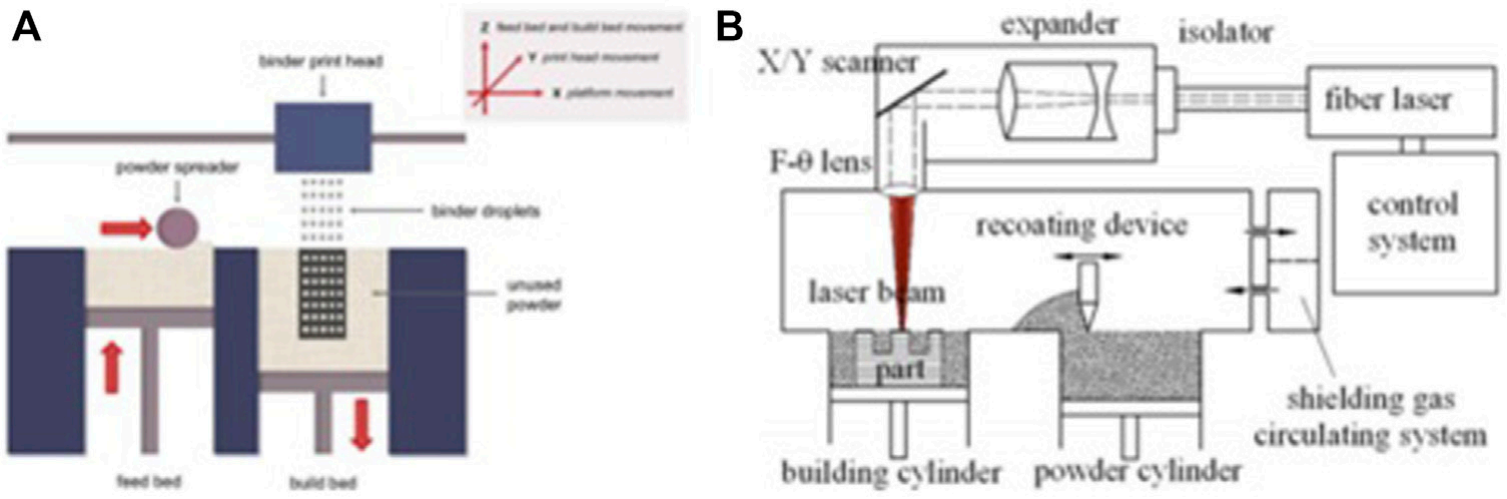
Schematics of the powder bed method **(A)** and the SLM method **(B)**, reproduced with the permission (Brunello et al., 2016) *Copyright © 2016 Elsevier Inc.* and the permission (Liu et al., 2015b) *Copyright © 2015 Published by Elsevier Ltd.*, respectively.

SLM and electron beam melting are the most extensively applied AM techniques for porous metal scaffolds because of their high precision, high efficiency, and excellent stability. The first layer of the metallic powder is applied and fused into a solid part by using a heat source of a laser or electron beam. When the construction platform drops, the next layer of powder is applied from the material distribution platform and fused. Once the whole structure is completed, the produced object is cut from the support body (Wang et al., 2017a). Panels A and B in Figure 3 present the schematics of the powder bed system and SLM, respectively.

By using the laser 3D printing method, Balla et al. (Krishna Balla et al., 2010) obtained porous Ta with 27% and 55% porosity. *In vitro* cell characterization showed that the biocompatibility of porous Ta is superior to that of porous Ti. The expression of alkaline phosphatase (ALP) of human fetal osteoblasts cultured on the porous Ta surface was higher, suggesting that Ta has higher osteogenic activity than Ti. Wauthle et al. (Wauthle et al., 2015b) obtained a porous Ta implant (porosity: 80%) and used it to repair large bone defects. The porous Ta had a yield strength of 12.7 MPa, a compressive strength of 36.1 MPa, an elastic modulus of 1.22 GPa, and a fatigue strength of approximately 7.35 MPa, thus meeting the criteria for a bone implant material. Porous Ta exhibited no cytotoxicity in vitro tests. Twelve weeks after implantation, bone ingrowth was observed. Complete bone contacts were observed in certain cases. The anti-torsion test showed that porous Ta has a strong ability to bind to the surrounding bone tissue. Dou (Dou et al., 2019) et al. prepared porous Ta scaffolds through CVD combined with chemical meteorological deposition. The *in vitro* test results indicated that the adhesion and proliferation of bone marrow mesenchymal stem cells (BMSCs) on porous Ta are significantly superior to those on porous Ti. Moreover, the ALP activity assay suggested that the osteogenic activity of porous Ta is higher than that of porous Ti. The q-PCR test showed higher expressions of osteogenic genes encoding ALP, osterix, collagen-I (Col-I), osteonectin, and osteocalcin (OCN) on porous Ta. The Western blotting assay revealed higher p-ERK protein expression on porous Ta. The aforementioned analyses suggested that porous Ta facilitates BMSC adhesion and proliferation and exhibits excellent osteogenic activity and osteoinductivity compared with widely used Ti and its alloys.

In brief, different pore formation mechanisms of the aforementioned approaches contribute to differences in the porous structure characteristics. The foam impregnation method is rarely used because of its excessive disadvantages such as pore plugging. The PM-manufactured scaffold has low pore interconnectivity, which results in limited osteoconductivity and bone penetration capacity. CVD generates trabecular-like pores with high interconnectivity, thereby promoting bone tissue ingrowth. CVD still lacks control of the porous structure. By contrast, AM-fabricated scaffolds exhibit high interconnectivity and controllable pore characteristics (strut diameter, pore diameter, pore geometry, and porosity). Highly customized Ta scaffolds with well-designed porous structures and anatomically matched geometry can be produced using AM, which makes AM the most extensively used and the most efficient approach for generating porous Ta implants. With advances in AM-related technologies, AM is expected to be the mainstream method for preparing porous Ta scaffolds.

## 4 Surface modification of porous Ta

The surface microenvironment is of great significance in orthopedic implants. Appropriate surface characteristics improve the area and quality of bone–implant contact, thereby facilitating osseointegration. Osseointegration is the direct connection between the bone and implant, which is critical to implant stability and for ensuring successful implantation (Turkyilmaz, 2011). However, osteointegration is a lengthy process, usually spanning weeks to months, and has four stages: hemostasis, inflammation, proliferation, and remodeling (Hendrik et al., 2012). Guglielmotti et al. (Guglielmotti María et al., 2000) conducted several experiments to determine the factors for osteointegration. The results showed that surfaces with higher roughness facilitate osseointegration. Hence, surface modification of implants is essential for enhancing the binding between the bone tissue and implant. Moreover, the corrosion resistance, wear resistance, and biocompatibility of the implants need to be improved (Sasikumar et al., 2019). Since the 1970s, studies have focused on the surface modifications of Ta and porous Ta to facilitate bone healing and offer more durable and stable bone binding. The current main surface modification techniques include biomimetic calcium phosphate coating, anodic oxidation, micro-arc oxidation (MAO), alkali–heat treatment, and surface functional modification.

### 4.1 *Alkali*–*heat treatment*

In alkali–heat treatment, the implant is soaked in NaOH solution and heated at a high temperature. During heating, biomimetic apatite is formed on the implant surface (Miyazaki et al., 2000). In the alkali treatment, the sodium salt gel is produced that weakens the bonding between the apatite and substrate and interferes with the bone–implant bonding. After the heat treatment, the gel dehydrates into a dense and stable amorphous structure, leading to a cohesive bonding between the apatite and substrate. Li et al. (Li et al., 2010a) suggested that the activated surface of the porous Ta alloy contributes to SaOS2 cell adhesion and spread. Kuo and colleagues (Kuo et al., 2019) recently used alkali–heat treatment to activate Ta. First, the sample was immersed in a 1 M NaOH aqueous solution at 60°C for 24 h, heated to 300°C at a rate of 5°C/min, and kept at this temperature for 1 h. The resulting mixture was cooled to room temperature. Subsequently, bone-like apatite was inducted through immersion in the simulated body fluid. This proved that the alkali–heat treatment accelerates the apatite growth rate. In addition, the *in vitro* test revealed better adhesion and spread of MG-63 cells cultured on alkali–heat-treated coating.

### 4.2 Anodic oxidation

In anodic oxidation, a potential between the anode (the metal) and the cathode (the electrode, e.g., graphite, platinum, and lead) is applied to transfer charges and ions and form an oxide layer. The process leads to the production of protective oxide layers with controlled thickness (Chang and Webster Thomas, 2006). When the voltage is applied, the anode surface undergoes oxidation, forming a dense oxide film. This film effectively inhibits the release of metal ions and improves corrosion resistance and bioactivity. Moreover, the adjustment of process parameters, including the electrolyte composition and concentration, as well as the temperature, applied potential, process duration, and power supply mode, regulates the morphology, roughness, and physical and chemical properties of the coating (Ercan et al., 2011; Minagar et al., 2012; Fialho and Carvalho, 2019). Anodic oxidation has been successfully used with titanium and other metals to produce ordered nanostructures, which promote the adsorption of proteins, ions, and cells (Wang et al., 2019a; Dias-Netipanyj et al., 2020).

**FIGURE 4:**
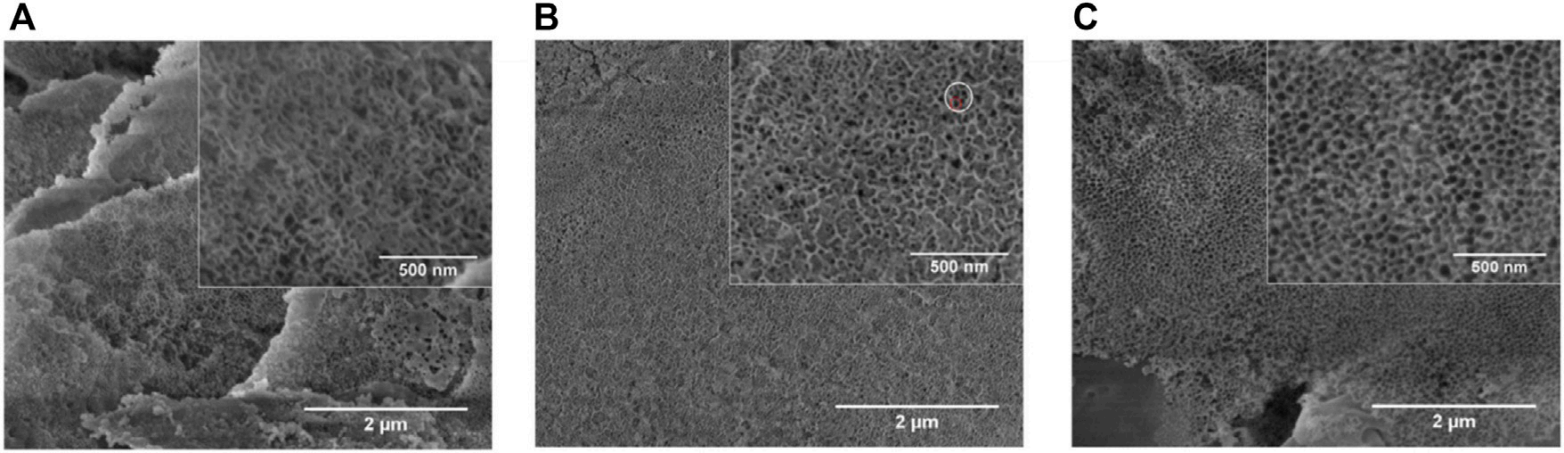
Scanning electron microscopy (SEM) images of Ta with different NH_4_F concentrations (Fialho et al., 2020). *Copyright ©2020 Published by Elsevier Ltd.*

L. Fialho et al. (Fialho et al., 2020) prepared a Ta_2_O_5_ nanoporous surface through anodic oxidation in an HF-free electrolyte composed of ethylene glycol, water, and ammonium fluoride (NH_4_F) with different anodic oxidation parameters (Figure 4). The parameters included electrolyte concentration, applied potential, and time. The samples with more uniform porous nanostructures were characterized in terms of their cross-sectional morphologies, chemical compositions, and crystal structures. The results proved that the Ta_2_O_5_ coating is well prepared on the porous Ta surface and facilitates cell adhesion. Ding et al. (Ding et al., 2018) prepared micro/nanostructures on Ta-coated surfaces by combining anodic oxidation and plasma spraying. The experimental results showed that the coating facilitates the proliferation, adhesion, and diffusion of human BMSCs, and the nanocoating increased gene expression by 1.5–2.1 times compared with the microporous Ta coating. An *in vitro* study (Wang et al., 2012) suggested that the modified Ta scaffold enhances rabbit BMSC adhesion and proliferation and upregulates the expression of osteogenic markers, ALP, Col-I, and OCN. Other *in vitro* experiments indicated that the anodic oxidation-modified Ta surface can facilitate the adhesion and proliferation of human osteoblasts, upregulate ALP expression in these osteoblasts, and promote bone nodule production and mineralization and deposition of the bone matrix (Frandsen et al., 2014).

### 4.3 Micro-arc oxidation

**FIGURE 5:**
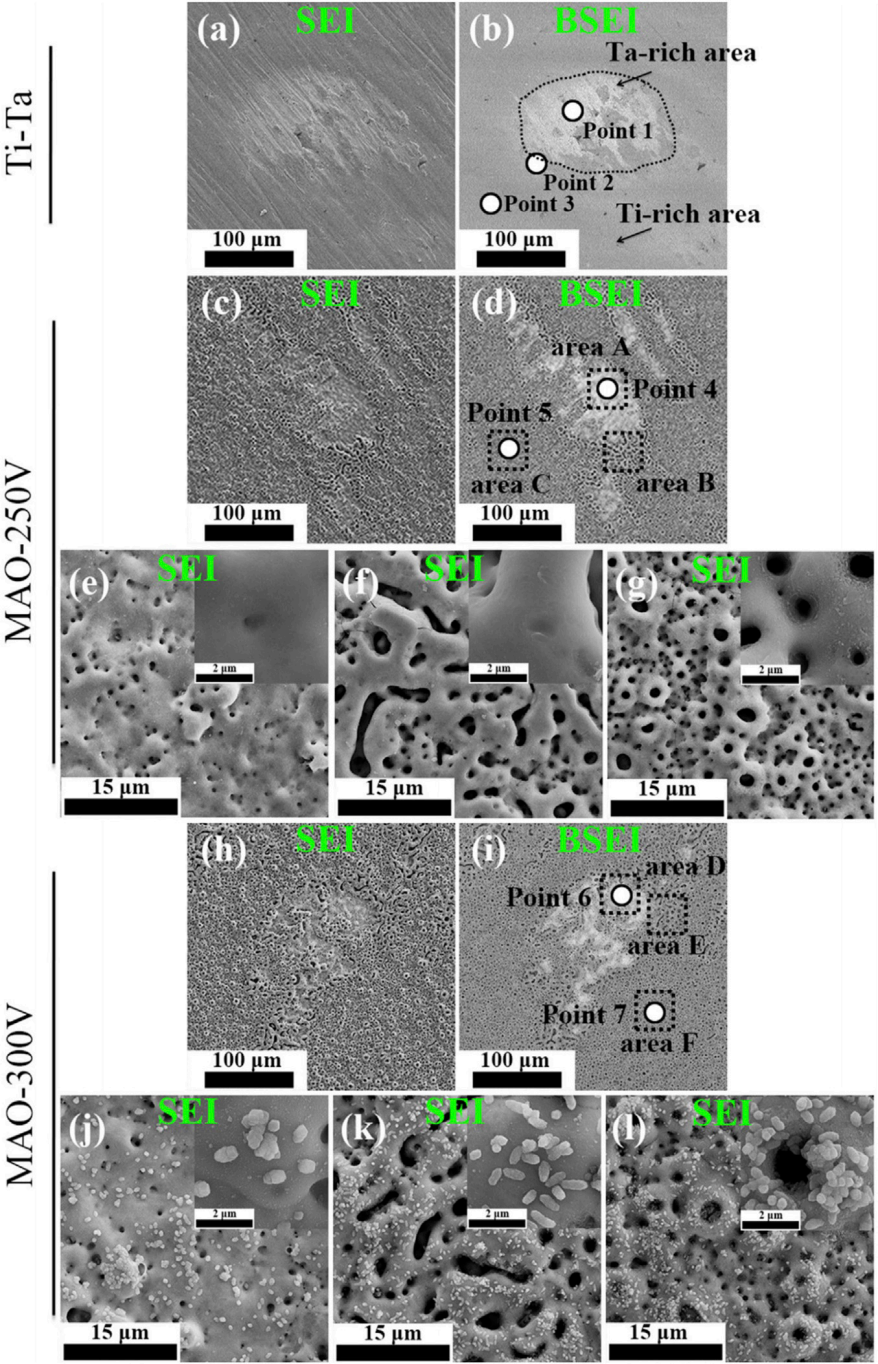
Secondary electron and backscattered electron SEM images showing the morphological features of various surfaces. Figures **(E–G)** and **(J–L)** correspond to areas **(A–F)** indicated by the black dashed lines in Figures **(D)** and **(I)**, respectively (Huang et al., 2018). *Copyright ©2018 Published by Elsevier Ltd.*

**FIGURE 6:**
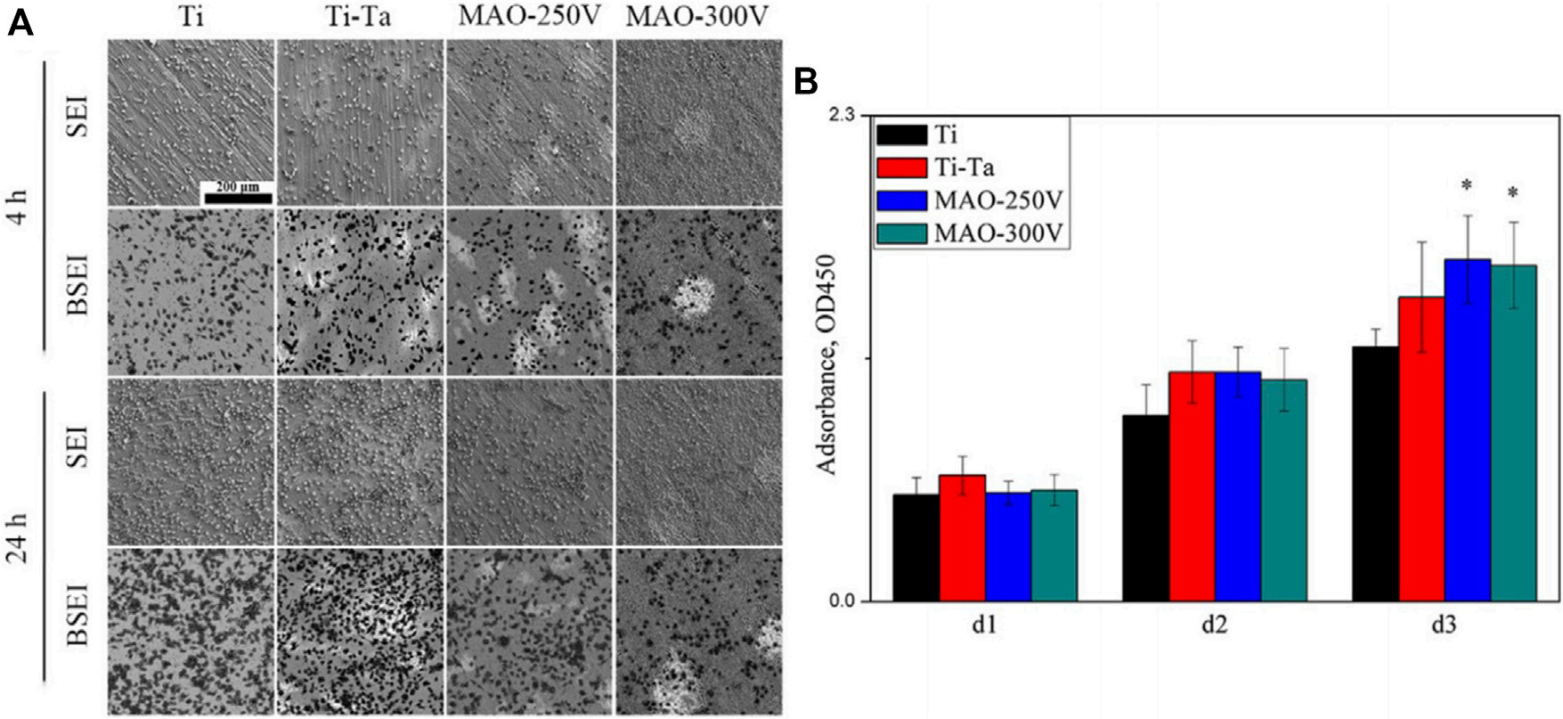
SEM images **(A)** and CCK-8 assay **(B)** showing the distribution and proliferation of SaOS-2 cells cultured on various surfaces. (Huang et al., 2018). *Copyright ©2018 Published by Elsevier Ltd.*

MAO, also known as plasma electrolytic oxidation, is an optimized version of anodic oxidation because of the formation of a ceramic coating mainly composed of substrate oxides and compounds containing electrolyte components (Kung et al., 2010). Studies have suggested that MAO coating incorporates bioactive elements (e.g., calcium, phosphorus) with certain electrolytes (Deng et al., 2010; Bai et al., 2011). MAO is superior to anodic oxidation in all aspects. The surface layer prepared by MAO has better corrosion resistance and wear resistance and cannot be easily separated. Gao et al. (Gao et al., 2014) employed MAO combined with alkali treatment to form an oxide coating on the porous Ta surface. *In vivo* and *in vitro* tests have confirmed that the coating enhances bioactivity *in vitro*, promotes angiogenesis and new bone formation, and facilitates new bone tissue infiltration into the scaffold. Huang et al. (Huang et al., 2018) used MAO to prepare oxide coating on the surface of Ti–Ta composites. The surface roughness and hydrophilicity of the treated area improved, which promoted the proliferation and differentiation of osteoblast-like SaOS-2 cells (Figures 5, 6).

### 4.4 Biomimetic calcium phosphate coating

Calcium phosphate coating has bone conductivity and promotes bone inward growth because of its physical and chemical similarities to human bones. Technological breakthroughs have recently been made in depositing the calcium phosphate layer on the substrate, such as electrochemical deposition, sol-gel, plasma immersion injection, and plasma spraying. Moreover, the formed crystals are more similar to natural bone minerals in structure than hydroxyapatite (HA) and tri-or tetra-calcium phosphate (Klein et al., 1994). The calcium phosphate biomimetic coating is more conducive to the differentiation of bone marrow stromal cells into osteoblasts than conventional coatings (Ohgushi and Caplan, 1999). Antonio et al. (Antonio et al., 2019) recently prepared a HA coating on a pure Ta surface through MAO. Compared with Ta coating, the HA coating significantly improved surface bioactivity. In addition, Ta_2_O_5_ added to the HA coating provides more calcium absorption sites and increases HA formation, thereby accelerating bone integration (Horandghadim et al., 2019).

### 4.5 Surface functional modification

Surface functionalization refers to modifying the implant surfaces by using growth factors, extracellular matrix proteins, peptides, and drugs to form surfaces with specific functions (García-Gareta et al., 2017). An ideal functional coating comprises uniformly incorporated natural or synthetic bioactive substances, such as bone morphogenetic proteins (BMPs), vascular endothelial growth factor, transforming growth factor, and antibiotics, which are stably released in a localized and controlled manner (Tobin, 2017). In this process, a minimum effective dose is essential for avoiding toxicity and side effects.

**FIGURE 7:**
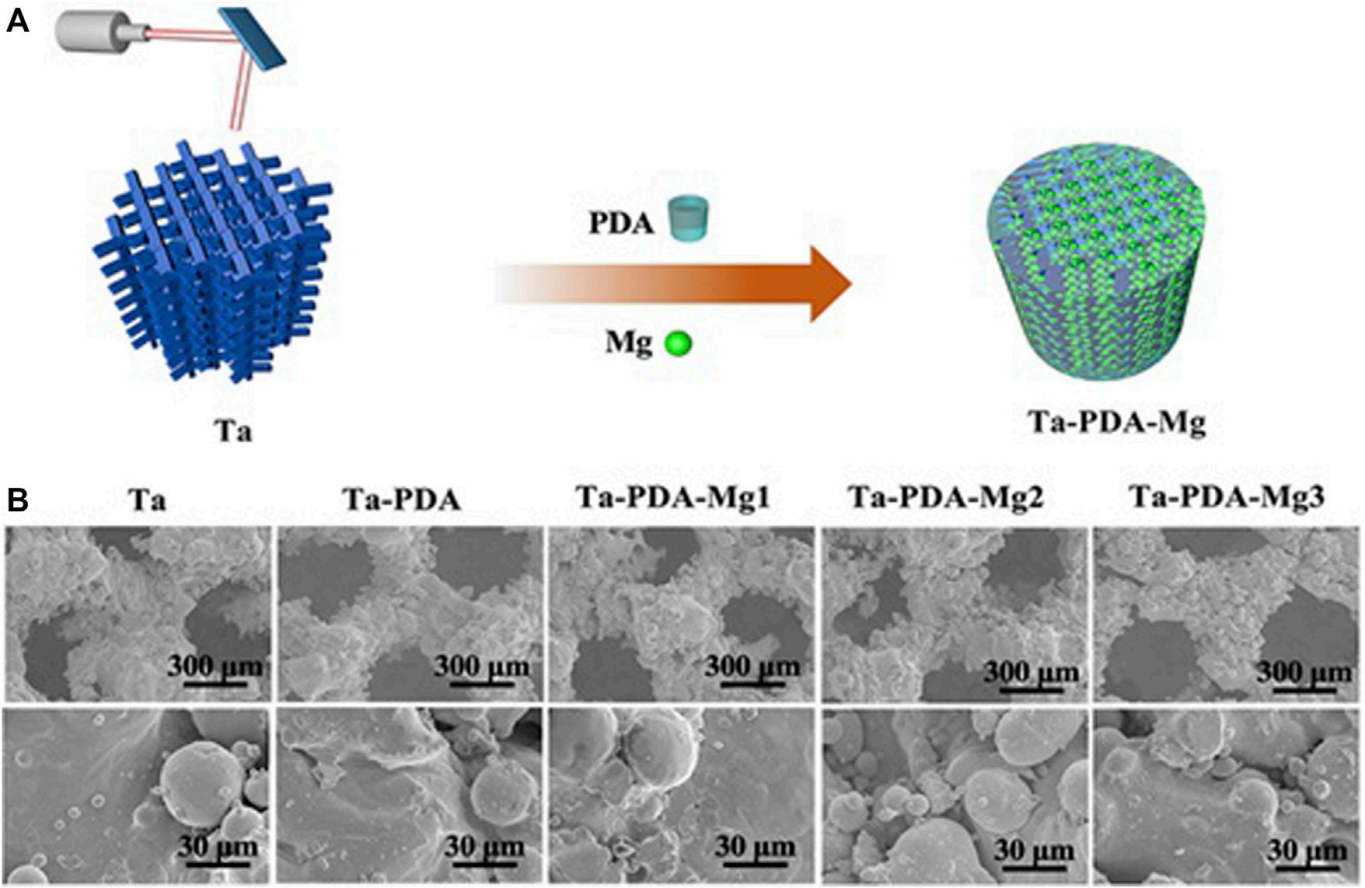
Schematics of Ta-PDA-Mg scaffold fabrication process **(A)**, and SEM images of the scaffold **(B)** (Ma et al., 2020b) *Copyright ©2020 Published by Elsevier Ltd.*

**FIGURE 8:**
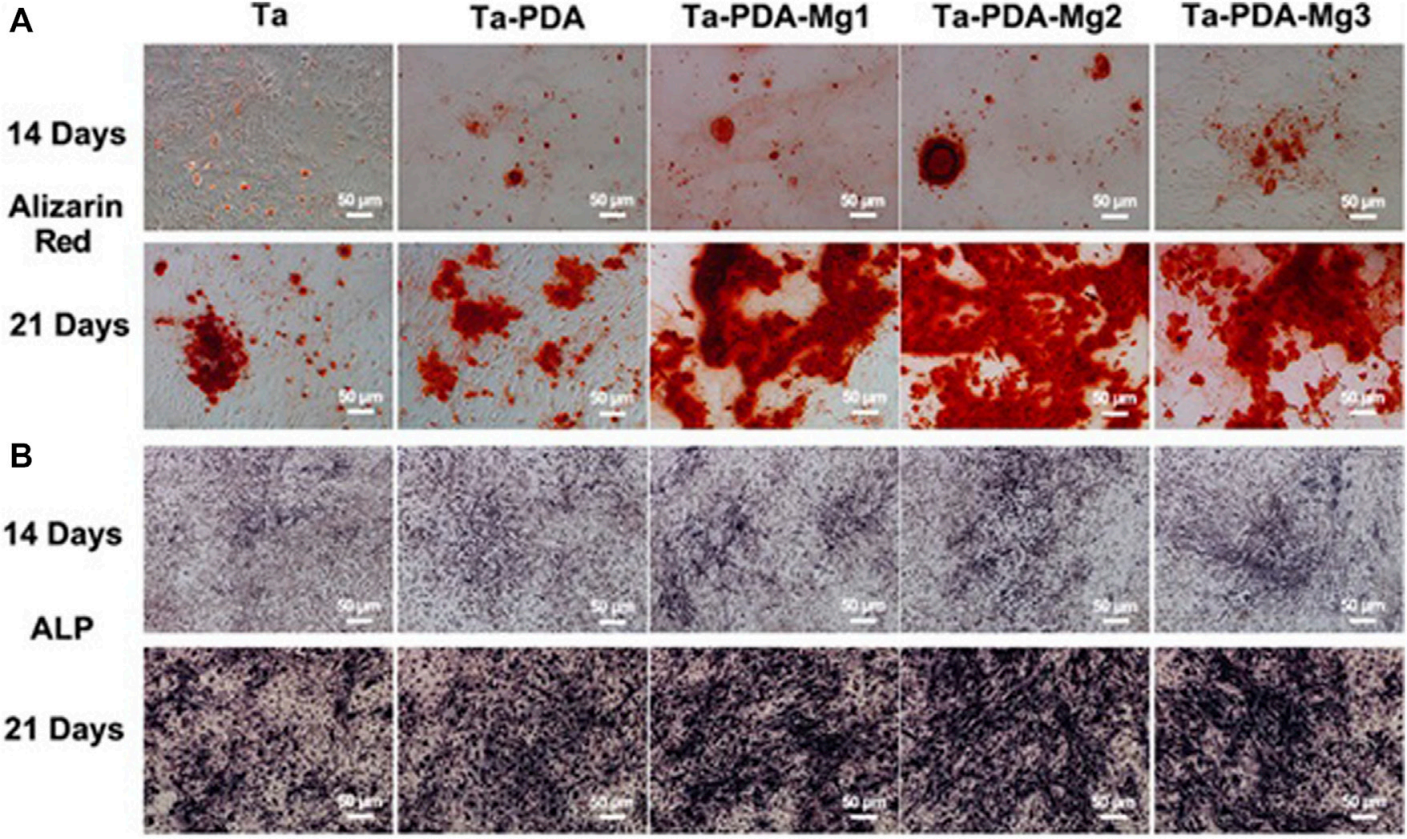
*In vitro* osteogenic evaluation of the Ta-PDA-Mg scaffold. Alizarin red staining **(A)** and ALP **(B)** of rBMSCs cultured with various extracts at 14 and 21 days (Ma et al., 2020b). *Copyright ©2020 Published by Elsevier Ltd.*

Wang et al. (Qian et al., 2018) used a rabbit osteochondral defect model of the femoral condyle and implanted BMP-7-coated porous Ta or uncoated porous Ta into the bone defect area. At 16 weeks after surgery, the micro-CT examination revealed that the new bone volume fraction and the quality and quantity of new bone trabeculae in the coated porous Ta group were superior to those in the uncoated group. Histological studies confirmed that more bone tissue was formed in the pores of the Ta scaffold in the coated group. The biomechanical analysis revealed that the maximum pullout force was significantly higher in the coated group than in the uncoated group. By building a 15-mm segmental defect model in the middle segment of the right radius of New Zealand white rabbits, Wang et al. (Wang et al., 2017b) verified the performance of an RGD peptide-coated porous Ta scaffold. The results indicated that bone formation at the interface and in the pores increased after application of the RGD peptide coating, thereby enhancing bone defect repair. Ma et al. (Ma et al., 2020b) used polydopamine (PDA) to load magnesium (Mg) on the surface of a porous 3D-printed Ta scaffold to enhance their surface bioactivity (Figure 7). The Ta-PDA-Mg scaffold released Mg ions and exhibited excellent biocompatibility (Figure 8). The Ta-PDA-Mg scaffold effectively promoted angiogenesis and osseointegration in a rat femoral condylar bone defect model. Through the formation of drug-doped self-assembled films, bioactive agents could be continuously released through the functionalized porous Ta surface. Garbuz et al. (Garbuz et al., 2008) designed a porous Ta scaffold for the local release of alendronate, which was fixed by immersing calcium phosphate-coated porous Ta in an alendronate buffer solution at room temperature for 7 days. During the whole release process, compared with the control group, the locally released alendronate inhibited osteoclast activity, resulting in an increase in osteoblast activity, and eventually, promoting the formation of more new bone on the scaffold surface.

Although surface modifications have been widely used and continually improved, their clinical applications have been rare. Many studies need to be conducted for their effective and reliable clinical translations.

## 5 Biological performance research

After implants are placed in the body, they remain in the body as a foreign body for a long time. The implant interacts with the specific biological environment in the body until equilibrium is attained or the implant is removed from the body. As a bone implant material, porous tantalum allows a large number of new bone tissues to grow into the implant, showing excellent bone integration performance. Numerous scientific studies have attempted to quantitatively evaluate the biocompatibility and osteogenic properties of porous tantalum through *in vitro* cytotoxicity assay, *in vivo* segmental bone defect model, and histological analysis.

### 5.1 Cytotoxicity studies

Tantalum has been reported to show low cytotoxicity *in vivo* or *in vitro*, tissue or cell implants of various shapes. Liu et al. (Liu et al., 2022a) used FDA/PI dye solution to dye live and dead cells on the scaffold surface, respectively, and observed and photographed them through CLSM; live cells stained green and dead cells stained red, and fewer dead cells (red) were observed. Li et al. (LI et al., 2010b) used osteoblast SaOS2 to evaluate the cytotoxicity of tantalum, titanium, niobium, molybdenum, niobium, and other common elements in titanium alloy and observed the cytotoxicity of these elements in powder and block. Cell experiments confirmed the cytotoxicity of titanium, niobium, molybdenum, and other metal powders, and molybdenum showed cytotoxicity in block. The safe ion concentrations of molybdenum, titanium and niobium were 8.5, 15.5, and 172.0 μg/L, respectively (a concentration lower than the safe ion concentration is non-toxic), but no obvious cell damage was observed in tantalum. Wauthle et al. (WAUTHLE et al., 2015) placed mouse fibroblasts in the extract of porous tantalum scaffolds for 41 h and conducted *in vitro* experiments to evaluate their cytotoxicity. The results showed good biocompatibility and no cytotoxicity of scaffolds. In addition, tantalum metal can be easily combined with oxygen to form an oxide layer (Ta_2_O_5_) on the surface of porous tantalum implants, which not only prevents corrosion of the implants *in vivo* but also ensures stability over a wide pH range (Wang et al., 2012). Cell morphology and cell activity are also indicators to evaluate cytotoxicity. Gee, etc. (GEE et al., 2019) evaluated the porous tantalum between human fibroblasts and osteoblasts and proliferation of mesenchymal stem cells (MSCs). *In vitro* studies have shown that porous tantalum exerts neither any significant negative effect on fibroblasts after 28 days of continuous culture nor any inhibitory effect on the proliferation and behavior of osteoblasts or human MSCs.

### 5.2 Osseogenesis study

Osseointegration is the direct integration of phalanges and metals that allows the structural and functional integration of the living bone on implant surface (GEE et al., 2019). The process of bone integration may be affected by various factors (WANG et al., 2016b), which can be divided into two aspects: the bone–implant interface environment and the implant itself. Environmental factors include loading conditions, host bone characteristics, interface distance, local osteoblast, osteoclast concentration, systemic disease (diabetes, rheumatoid arthritis), and smoking (WANG et al., 2018), whereas implant factors include geometry, surface topography, and line design (KOBAYASHI et al., 2016). In general, tantalum metal is bioinert, that is, it does not stimulate bone growth (CHEN et al., 2012). However, porous tantalum has been shown to have satisfactory bone integration and bone conductivity. It allows the proliferation and differentiation of osteoblasts and promotes the growth of bones, tendons, and ligaments. Wei et al. (SHI et al., 2017) implanted porous tantalum rods into the hind legs of dogs. Three to 6 weeks after implantation, new osteoblast adhesion and new bone ingrosion were observed at the tantalum–host bone interface and pores through hard tissue biopsy. Fraser et al. (FRASER et al., 2019) implanted a dental implant with a titanium neck and root tip connected to an intermediate portion made of porous tantalum in a rabbit tibial repair model. They found that the middle part was in close contact with the surrounding soft and hard tissues. Studies have shown that tantalum surface properties affect the implant–bone interface microenvironment and play a crucial role in osteoblast proliferation and reconstruction as well as interfacial bone integration.

**FIGURE 9:**
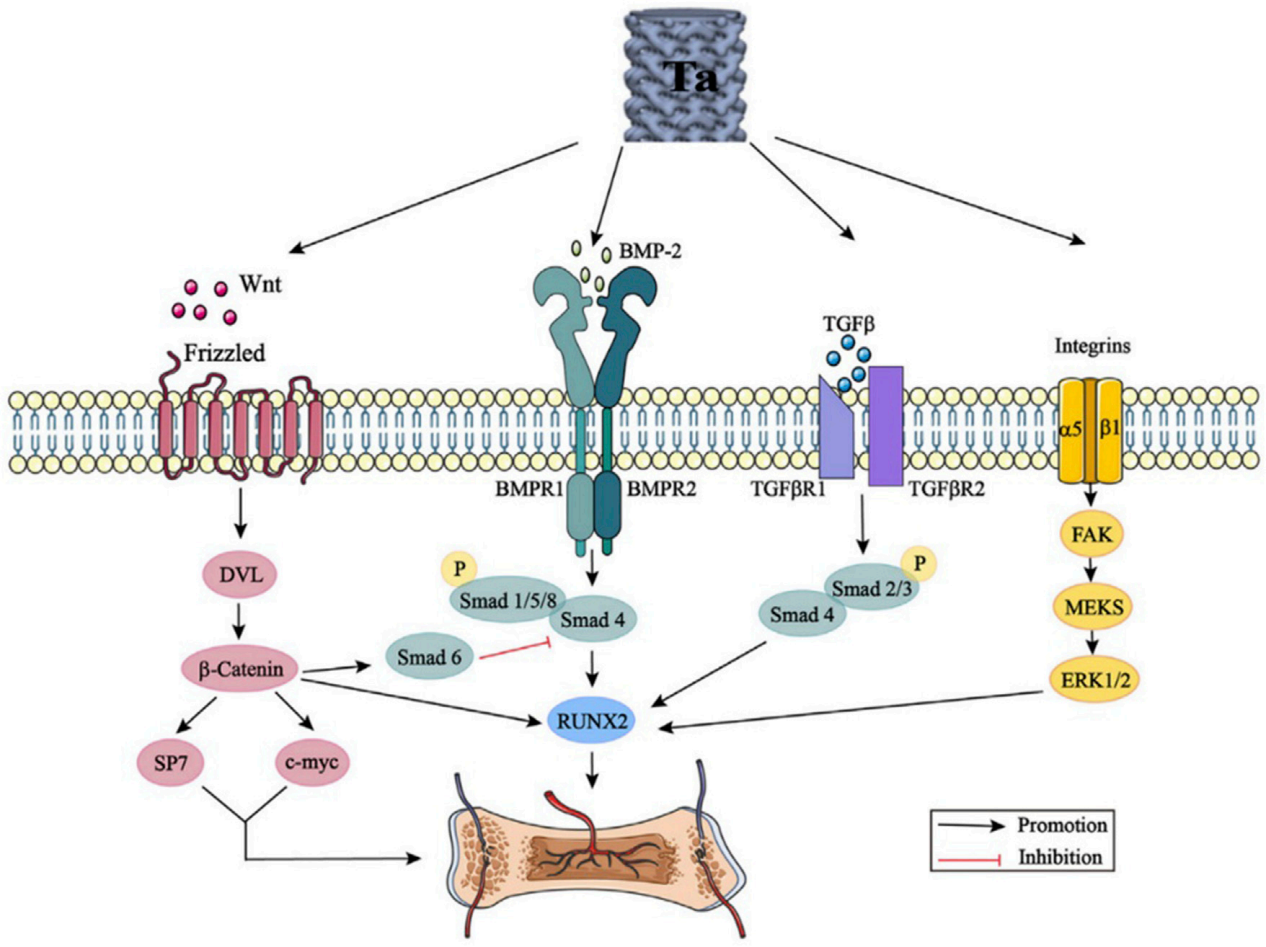
Tantalum activates the Wnt/β-catenin signaling pathway, BMP signaling pathway, TGF-β signaling pathway, and integrin signaling pathway by promoting the expression of Wnt, BMP-2, TGF-β, and integrins. DVL: disheveled; BMP: bone morphogenic proteins; Smad: small mother against decapentaplegic; Runx2: runt-related transcription factor 2; TGF-β: transforming growth factor-beta; FAK: focal adhesion kinase; and ERK: extracellular signal regulated kinase (Wang et al., 2022). From Wang et al., 2022 (Figure 3)

Many studies have attempted to explain the action mechanism of tantalum in bone tissue growth through the osteogenic signaling pathways including Wnt//β-catenin signaling pathway, BMP signaling pathway, TGF-β signaling pathway, and integrin signaling pathway (Figure 9). However, there are only a few biological studies and known mechanisms related to porous tantalum, despite complex crosstalk effects between these signaling pathways. More comprehensive, detailed, and in-depth mechanistic studies need to be conducted in the future.

## 6 Clinical applications

Over the past decades, porous Ta implants have gained considerable attention, especially in the clinical orthopedics field. Porous metals applied in clinical practice mainly refer to trabecular metal (TM) produced by Zimmer (Minneapolis, MN, United States). TM is composed of porous Ta. Porous Ta scaffolds have been extensively used for clinical orthopedic applications, including the hip joint, knee joint, spine, foot and ankle joints, and oral cavity.

### 6.1 Hip joint

#### 6.1.1 Femur head osteonecrosis

Necrosis of the femoral head (ONFH) is a pathological state, wherein the blood supply of the subchondral bone is reduced. Changes in bone trabecular structures and articular surface collapse are caused by multiple factors. According to the Steinberg classification, ONFH is divided into five phases. In Phase I and Phase II, when the non-operative treatment fails, doctors prefer core decompression with Ta TM rod implantation. Core decompression has long been a hip preservation treatment for early ONFH, whereas the lack of mechanical support for subchondral bone after necrotic bone debridement may lead to femoral head collapse (SCULLY Sean and Urbaniak, 1998). Thus, porous Ta rods, as a plausible substitute for the vascularized autofibular allograft, have allowed the maintenance of the bone defect portion after core decompression (Tsao et al., 2005; Wei et al., 2019).

Porous Ta rods are primarily used to maintain the subchondral plate structure and stimulate host bone osteogenesis, which can alleviate ONFH deterioration and delay the final conversion to total hip arthroplasty (THA), as indicated in most studies conducted in early- or middle-stage patients (Veillette et al., 2006; Shuler et al., 2007; Liu et al., 2010).

Although the long-term efficacy of Ta rods remains controversial, patient survival after the insertion of porous Ta rods is known to be affected by multiple factors such as disease stage, corticosteroid use, volume and location of osteonecrosis lesions, bone marrow edema, and joint effusion (Zhang et al., 2016; Zhang et al., 2021; Liu et al., 2022b). Accordingly, numerous optimized surgical techniques have been applied to improve the osteogenic capacity of porous Ta rods such as the combined technique involving bone marrow extraction from the iliac crest (Emilios et al., 2015), BMSCs, and graft (Pakos et al., 2015; Zhao et al., 2015). Nevertheless, long-term follow-up clinical trials are required to validate the effectiveness of the aforementioned modification methods.

#### 6.1.2 Acetabular components of porous metals

**FIGURE 10:**
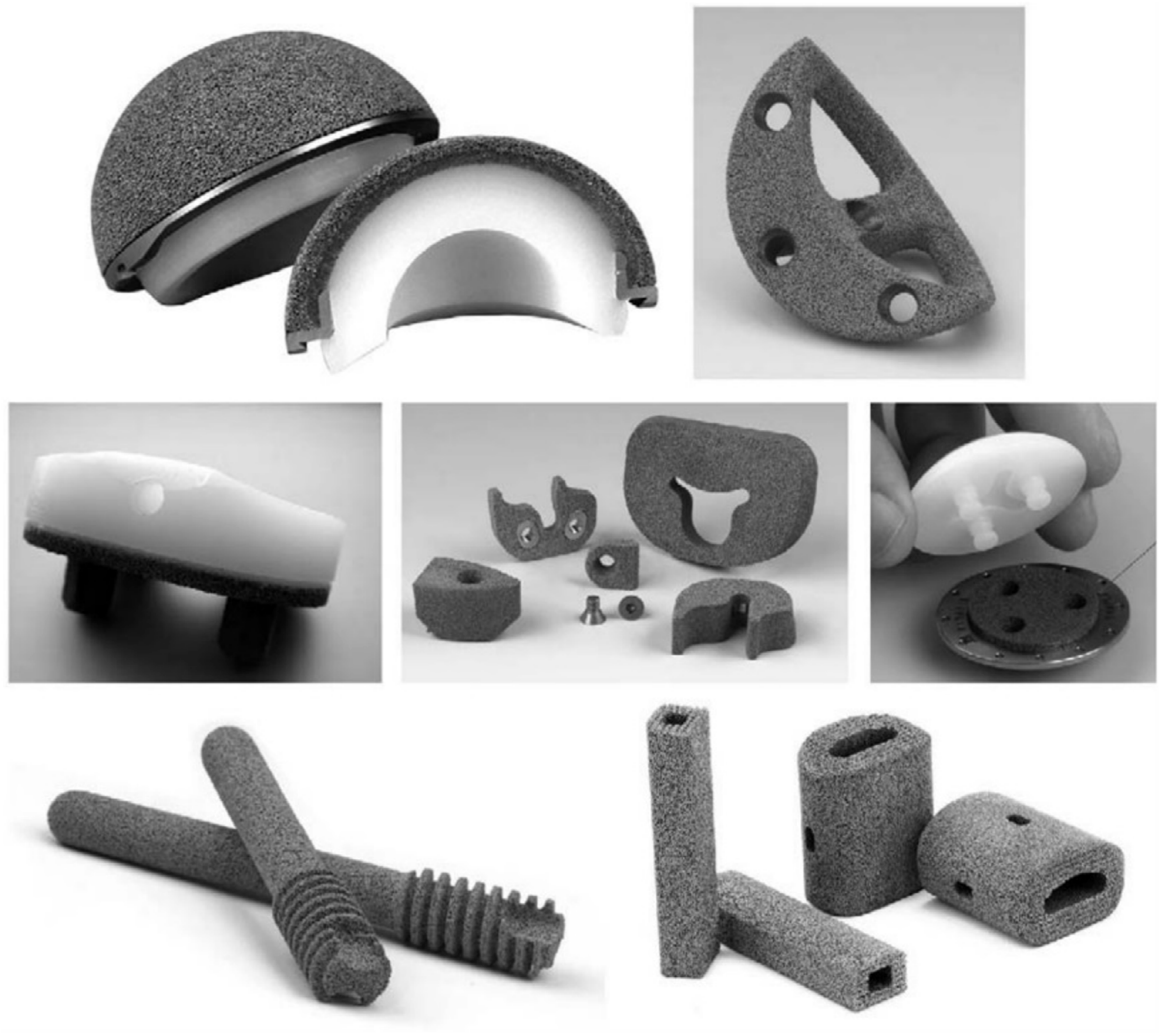
Multiple orthopedic applications for porous tantalum. Top row: monoblock acetabulum and a revision acetabular augment. Middle row: monoblock tibia, revision total knee arthroplasty augments, and salvage patella button. Bottom row: osteonecrosis implant and spine arthrodesis implants (Courtesy of Zimmer, Warsaw, IN) (Russell Levine et al., 2006). *Copyright ©2006 Published by Elsevier Ltd.*

Acetabular cups made of porous Ta that are used for primary THA are assembled by compressing ultra-high molecular polyethylene into elliptical porous Ta shells. For THA revision, modular and revision multi-hole porous Ta shells are available (Figure 10). Owing to their low elastic modulus, high friction coefficient, and superior osteoconductivity, porous Ta shells are beneficial for maintaining or even increasing the bone mass of the adjacent acetabulum and contribute to revision surgery, when needed (Vutescu et al., 2017; Beckmann et al., 2020). In a prospective study (Macheras et al., 2008) with 8–10 years of follow-up of 151 hips after primary THA, although the periacetabular gaps of length 1–5 mm were found in the early 25 acetabular cups, the aforementioned gaps disappeared after 24 weeks. Follow-up X-ray images confirmed the absence of light transmittance, osteolysis of the adjacent bone, polyethylene wear debris, and cup loosening. The aforementioned analyses verified the advantage of porous Ta cups in design and their clinical efficacy.

In a prospective clinical study (Duan et al., 2021) on the clinical application of Ta, eight patients (two male and six female patients, 12 hips, mean age: 43.75 ± 7.81 years) with Crowe I developmental dysplasia of the hip (DDH) were subjected to hip reconstruction by using an additional customized porous Ta acetabular patch. After computerized modeling of the patients’ hip joint separately, a software was used to design the best acetabular patch, and then, the finite element analysis was performed to ensure biomechanical requirements. Finally, an AM-generated customized and personalized porous Ta acetabular patch was implanted. After an average follow-up of 8.2 months, the visual analog score (VAS) decreased significantly from 2.92 ± 0.79 before the operation to 0.83 ± 0.72 after the operation, and the Harris score decreased significantly from 69.67 ± 4.62 before the operation to 84.25 ± 4.14 after the operation. Imaging revealed that the acetabular patch was in close contact with the iliac bone, and no loosening or progression of osteoarthritis occurred. Therefore, the application of AM-produced individualized porous Ta patch for reconstructing acetabular bone defects can reduce the difficulty of surgery and delay osteoarthritis progression.

### 6.2 Knee joint replacement

Porous Ta prostheses for knee replacement comprise single tibial components, tibial or femoral cones and prostheses, and patellar prostheses (Huang et al., 2021). The mechanical and biological properties of the porous Ta help achieve the primary stability of the tibial component and the patients’ long-term survival. Short-term and long-term results suggest that the cemented or non-cemented single tibial assembly has high efficacy in relatively young and active patients (Ivan et al., 2016; Defrancesco et al., 2018; De-Wei et al., 2019). Histological examination of the porous Ta tibial component removed from a chronically infected knee prosthesis revealed significant inward bone outgrowth at the pile and pile–substrate interfaces (rather than the substrate), which suggested remarkable bone–implant integration even in an infected environment (Sambaziotis et al., 2012; Peter et al., 2018).

### 6.3 Spinal surgery

In cervical and lumbar surgery, porous Ta has been successfully applied as an effective intervertebral device because of its unique biomechanical properties (Marko et al., 2016).

A prospective randomized controlled clinical trial (Fernandez-Fairen et al., 2008) confirmed the efficacy of a porous Ta cage for anterior cervical fusion. Compared with the conventional autogenous iliac bone transplantation in combination with the anterior plate, the porous Ta cage exhibited a considerable fusion rate (89% vs. 85%) and postoperative stability at 2-year follow-up, without additional fixation and graft harvest-related damage. At 11-year follow-up, although the implant settlement ranged from 2 to 3 mm, the clinical and radiological results of patients who received a single-hole Ta inter body fusion cage for inter body fusion were satisfactory, and 12 patients exhibited no obvious complications. Furthermore, several observational studies have confirmed that porous Ta is efficacious in terms of the intervertebral fusion rate, low complication rate, and improved short-term or long-term postoperative assessment scores, including SF-36, neck disability index, and VAS (Papacci et al., 2016; Rissmann, 1055; Wen-Qiang et al., 2019; Wang et al., 2019b).

### 6.4 Shoulder replacement

Zimmer launched three types of TM implants for shoulder replacement. X-ray images of at least 2 years of one-stage shoulder replacement with porous Ta prosthesis for the treatment of complex proximal humeral fractures were satisfactory (Li and Jiang, 2013). TM prosthesis demonstrated a better healing effect than traditional prostheses in a hemi joint replacement. The first-generation glenoid components (Zimmer) were introduced for total shoulder arthroplasty in 2003 but were withdrawn after the reports of prosthesis failures in 2005. In 2009, the second-generation glenoid components with a changed design were introduced. The satisfactory function, subjective satisfaction, and clinical manifestation of 3-year average follow-up were reported (Merolla et al., 2016). Porous Ta is not omnipotent, and complications should be avoided based on the clinical experience. In addition to bone growth, antibacterial performance is crucial for porous implants both in the short and long term. Thus, future studies should pay more attention to the development of implants with antibacterial properties.

### 6.5 Fractures

Internal fixation materials used for clinical fracture treatment usually involve high-strength stainless steel and Ti alloys. Using Ta-coated Ti6Al4V bone plates, Liu et al. (Liu et al., 2021b) treated tibia fractures in goats. The Ta-coated bone plates effectively fixed the fractures. Histologically, the new bone was formed at the interface with excellent osseointegration with the host bone.

A randomized controlled trial (Zhao et al., 2022) compared the clinical efficacy of cannulated compression screws and pressurized porous tantalum screws (PTS) in femoral neck fracture (FNF) treatment. The PTS exhibited a higher fusion rate and better postoperative stability than the cannulated compression screws at the 3-month follow-up. The fixation PTS for FNF at the center could avoid blood supply destruction in the femoral head, reduced the incidence of FNF postoperative complications, induced early bone ingrowth, and promoted fracture healing.

### 6.6 Foot and ankle surgery

The porous Ta spacer, considered a promising alternative to a conventional autograft or bone allograft, has been used for ankle arthrodesis without limitation by size, volume, and origin (Sagherian and Claridge, 2019; Tiusanen et al., 2019). Sundet et al. (Ms et al., 2021) used retrograde intramedullary nails, porous Ta spacers, and bone induction pads combined with an autologous bone marrow concentrate to perform revision surgeries on 30 patients (31 ankles) with failed total ankle arthroplasties. The mean fusion rate at the mean 23-month follow-up was 93. Most patients expressed satisfaction with the procedure in terms of pain relief and activity improvement. Kreulen et al. (Kreulen et al., 2017) introduced a novel strategy for reconstructive surgery in two patients with failed total ankle replacements and four patients with ankle collapses after infection. They introduced the porous Ta spacer to autologous bone marrow obtained from the femoral bone marrow cavity by using a reamer/irrigator/aspirator and fixed it with a tibial calcaneal nail and then supplemented with BMP-2 or platelet-derived growth factor to facilitate bone fusion. Using this novel approach, a thorough bone fusion occurred at the bone–implant interface in the early stages of 4–6 weeks after surgery, and no failed cases were reported.

### 6.7 Oral cavity

Rough surfaces extensively employed in dental implants facilitate osseointegration and angiogenesis by increasing the surface energy, expanding the bone–implant contact area, improving surface hydrophilicity, and promoting the adhesion of mesenchymal cells or osteoblast progenitors (Maxim et al., 2014; Bencharit et al., 2019). Porous Ta is superior to several other metals in bone growth and bone–implant contact. A clinical study (Brauner et al., 2015) suggests that dental implants made using porous Ta TM (PTTM) are safe and effective and cause no serious complications. Edelmann et al. (Edelmann et al., 2018) compared the peri-implant bone remodeling of PTTM-enhanced titanium implants and conventional titanium alloy implants in the first year after implantation. They found that the patients with PTTM-enhancing implants had lower bone loss rates than the controls (David et al., 2019; Piglionico et al., 2020).

## 7 Future development

Tantalum possesses extremely stable physical and chemical properties, in addition to excellent biocompatibility and other properties. Tantalum is one of the most promising bone graft replacement materials and has been increasingly favored by researchers. Bone implants made of porous tantalum materials are currently used in the treatment of bone defects in various parts of the body, and the short-term follow-up results also show relatively ideal clinical effects. However, as a material implanted in patients for a long time, it has some limitations. Combined with the research progress at home and abroad, we acknowledge the following problems:

(1) Porous tantalum is an inert material that cannot provide growth factors. Hence, surface modification studies are needed to determine whether it can be applied in combination with growth factors. Attempts can also be made to load antibiotics into the porous structure or to establish composite systems on the surface to prevent or treat infection of the joint around the implant.
(2) Tantalum is relatively rare, expensive, and difficult to process, which limit its clinical application. Therefore, breaking through the bottleneck of processing technology, reducing the development cost, and exploiting the excellent performance of porous tantalum implant materials for medical use are major research directions.
(3) The mechanical properties of porous tantalum can be matched with those of bone tissues by optimizing the design of the internal structure.
(4) Tantalum and porous tantalum surface modification technology is in a stage of rapid development. With the continuous optimization of this technology, its clinical application is expected to increase greatly. Presently, porous tantalum coating applied in clinic has not reached the ideal state, and long-term clinical follow-up data of large samples and multi-centers are lacking; hence, conducting a comprehensive evaluation of the safety and effectiveness of porous tantalum bone implants is challenging.

## 8 Summary

This systematic review elaborates on the preparation techniques, surface modification, and orthopedic applications of porous Ta scaffolds. Porous Ta scaffolds manufactured using different techniques exhibit excellent corrosion resistance, biocompatibility, osseointegration, and osteoconductivity, which highlight their great potential as orthopedic implants. The preparation and surface modification techniques of these scaffolds have been the focus of research.

CVD, an early manufacturing approach, is a well-established technique for manufacturing porous Ta scaffolds. Porous Ta implants in clinics are fabricated using this method. However, the shape of bone defects varies among patients depending on the anatomical sites and other conditions. Commercial CVD-manufactured porous Ta implants usually do not meet personalized requirements. An emerging technique, AM, can tailor patient-specific implants. AM-produced Ta implants are expected to be extensively used in orthopedic surgery. Regarding the modification of porous Ta, various methods (e.g., surface chemical modification and surface functionalization modification) have been developed to improve bioactivity. Modified porous Ta exhibits great potential in resolving various pathological conditions (e.g., osteoporosis, infection, diabetes, and even tumors).

Additional studies are required to explore the potential of porous Ta. With the booming evolution of porous Ta fabrication techniques, the clinical application of porous Ta is expected to expand in the future.
